# Flame Retardancy and Thermal Property of Environment-Friendly Poly(lactic acid) Composites Based on Banana Peel Powder

**DOI:** 10.3390/ma15175977

**Published:** 2022-08-29

**Authors:** Fanbei Kong, Baisheng Nie, Chao Han, Dan Zhao, Yanan Hou, Yuxuan Xu

**Affiliations:** 1 School of Resource and Safety Engineering, China University of Mining & Technology (Beijing), Beijing 100083, China; 2 State Key Laboratory of Explosion Science and Technology, Beijing Institute of Technology, Beijing 100081, China; 3 Dynamics and Control, School of Resources and Safety Engineering, Chongqing University, Chongqing 400044, China; 4 School of Energy Resources and Safety, Anhui University of Science and Technology, Huainan 232001, China

**Keywords:** banana peel powder, flame retardancy, poly(lactic acid), thermal property

## Abstract

Banana peel powder (BPP) was used to prepare poly(lactic acid) (PLA) bio-based composites and the flame retardancy was enhanced by introducing silica-gel microencapsulated ammonium polyphosphate (MCAPP). The results showed that the limiting oxygen index (LOI) of PLA containing 15 wt % BPP was 22.1% and just passed the UL-94 V-2 rate. Moreover, with the introduction of 5 wt % MCAPP and 15 wt % BPP, the PLA composite had a higher LOI value of 31.5%, and reached the UL-94 V-0 rating, with self-extinguishing and anti-dripping abilities. The PLA/M5B15 also had a lower peak heat release rate (296.7 kW·m^−2^), which was 16% lower than that of the PLA/B15 composite. Furthermore, the synergistic effects between MCAPP and BPP impart better thermal stability to PLA composites. According to the investigation of the char residue and pyrolysis gaseous products, MCAPP with BPP addition is beneficial to the formation of a higher quality char layer in the solid phase but also plays the flame retardant effect in the gas phase. This work provides a simple and efficient method to solve the high cost and flammability issues of PLA composites.

## 1. Introduction

Poly(lactic acid) (PLA) is a thermoplastic that shows the combined advantages of biodegradability, biocompatibility, and recyclability [1,2]. It is regarded as a promising candidate for petroleum-based plastics, and has been used in food packaging, disposable utensils, and medical devices [3,4,5]. However, the drawbacks of high cost, flammability and dripping combustion of PLA limit its widespread use [6,7,8]. To extend the application range of PLA, the strategies of combining PLA and cheap natural additives have been undertaken and developed in the field of composites. The effect of kraft lignin (KL) as a bio-based carbonization agent on the flame retardancy of PLA was investigated by Maqsood et al. [9]. The presence of 20 wt % ammonium polyphosphate (APP) enhanced the limiting oxygen index (LOI) of PLA/APP20 to 33.5%, and the LOI of the composite was increased to 34.4% when adding 5 wt % pentaerythritol (PER) as carbonization agent. Nevertheless, all these composites can only reach vertical combustion test (UL-94) V-1 rating. By comparison, the LOI of PLA/APP20/KL5 was further increased to 37.8% and passed the highest level of UL-94 V-0 rating, with anti-dripping properties. The results indicated that the bio-based carbonization agent KL had better char-promoting effect than traditional carbonization agents PER. Similar results have been obtained by other researchers, who worked on using biomass material as a bio-based carbonization agent to reinforce the flame retardancy of PLA. Yang et al. prepared PLA bio-composites by melt blending with oxidized wood flour (OWF), polyethylene glycol (PEG), and APP, which respectively acted as carbon source, plasticizer, and acid source [10]. In their discussion, they noted that the introduction of OWF and APP resulted in favorable flame retardant properties, with the composite achieving an LOI value of 30.6% and passing the UL-94 V-0 rating. Woo et al. carried out a twin-screw extrusion technique to prepare kenaf/PLA composites, and found that the flame retardancy of the composite was significantly influenced by the existence of APP and kenaf [11]. The addition of 30 wt % APP and 40 wt % kenaf increased the char yield of the composite, and reached an LOI value of 45.3%. In the works of Shukor et al. [12], thermogravimetric analysis (TG) indicated that the incorporation of 25 wt % kenaf and 20 wt % APP in PLA matrix led to a lower reduction decomposition temperature, but an increase in char residue at high temperature compared to the PLA/kenaf/PEG bio-composite.

Recently, banana peel powder (BPP), which is mainly composed of cellulose (7–10%), hemicellulose (6–8%), and lignin (6–12%), has been arousing more and more attention as a novel natural additive in composites [13,14]. Compared with other natural additives like wood flour [15], kenaf [16], ramie [17], bamboo [18], and starch [19], BPP as a product from agricultural wastes does not need extra planting area. Each year around 30 million tons of banana peel wastes are produced worldwide, which makes BPP an abundant resource at a cheap cost [20,21]. Meanwhile, the utilization of these agricultural wastes will reduce the environmental problems caused by improper treatments [22]. Furthermore, the good thermal stability and high fixed carbon content of BPP (almost 45 wt %) make it possible to be utilized as a bio-based carbonization agent [23,24,25]. Based on the above advantages of BPP, it has been considered as a suitable natural additive to prepare PLA/BPP composites. However, the flammable property of PLA/BPP limits its application. As far as we know, there are few reports on preparing flame retardant PLA/BPP composites [26,27].

Microencapsulation technology has been employed to effectively ameliorate compatibility in the APP and PLA matrix, resulting in the improvement of flame retardancy of the composites [1,28,29]. In this work, PLA/BPP composites were successfully prepared by the melt blending method, and silica-gel microencapsulated ammonium polyphosphate (MCAPP) was introduced as a flame retardant to enhance the flame retardancy of these composites. Then the flame retardancy and thermal properties of composites were investigated. The findings of this work can not only provide a simple method to improve the flame retardancy of PLA at a low cost, but also offer a novel way to use agricultural wastes such as banana peel.

## 2. Materials and Methods

PLA (REVODE190) consists of 97% L-lactic acid that was supplied by Zhejiang Hisun Biomaterials Co., Ltd. (Taizhou, China). BPP (particle size: 150 μm) was purchased from Shanxi Hengling Natural Biological Products Co., Ltd. (Xian, China). MCAPP (particle size: 16 μm, P_2_O_5_: > 70%) was bought from Zhenjiang XingXing Flame Retardant Co., Ltd. (Zhenjiang, China).

All materials were dried at 80 °C for 48 h before use. PLA pellets were melted via a twin-roller mill (XSS-300, Shanghai Chuangke Co., Shanghai, China) at 160 °C with the roller speed at 30 rpm for 5 min. Then BPP, MCAPP, and their mixture were gradually added according to the formulations with continuous blending at 30 rpm for 10 min. Next, the samples were hot-pressed at about 160 °C for 20 min. The formulations are listed in Table 1.

The LOI values were determined according to the GB/T 2406.2-2009 standard, and the instrument applied was an HC-2 oxygen index meter (Jiangning Analysis Instrument Co., Nanjing, China). The specimen dimension used for testing was 100 × 6.5 × 3 mm^3^, each sample was tested 5 times and the results were averaged to obtain the final LOI value. The UL-94 was measured by a CFZ-2-type instrument (Jiangning Analysis Instrument Co., Nanjing, China) according to the GB/T 2408-2008 standard with the dimension of the sample sheets of 100 × 13 × 3 mm^3^. A set of samples included 5 specimens and was tested 5 times; the specific determination of UL-94 rating is shown in Table 2. 

TG curves were performed on TGA/SDTA851e (Mettler-Toledo, Greifensee, Switzerland) instrument under air atmosphere. The specimen was heated from ambient temperature to 550 °C at a heating rate of 20 °C min^−1^. Cone calorimeter test (CCT) was carried out using a cone calorimeter (TTech-GBT16172, TESTech (Suzhou) Instrument Technologies Co., Suzhou, China) at the heat flux of 35 kW·m^−2^ with the dimension of the sample sheets of 100 × 100 × 3 mm^3^, according to the ISO 5660-1 standard. 

Scanning electron microscopy (SEM) was performed on the morphology of the char residues, using a kyky-2800B SEM with an accelerated voltage of 3 kV and 30 kV. Before the examination, all the non-conductive samples were sputter-coated by a thin layer of gold. 

Thermogravimetric analysis-infrared spectrometry (TG-IR) (DT-50, Setaram Instrument Co., Caluire, France) which is linked to IRAffinity-1 FTIR spectrometer was applied to study the pyrolysis volatiles of composites. The samples (around 10.0 mg) were heated from 30 to 700 °C at the rate of 20 °C·min^−1^ (air atmosphere).

## 3. Results and Discussion

### 3.1. Flame Retardancy of PLA Composites

**Figure 1:**
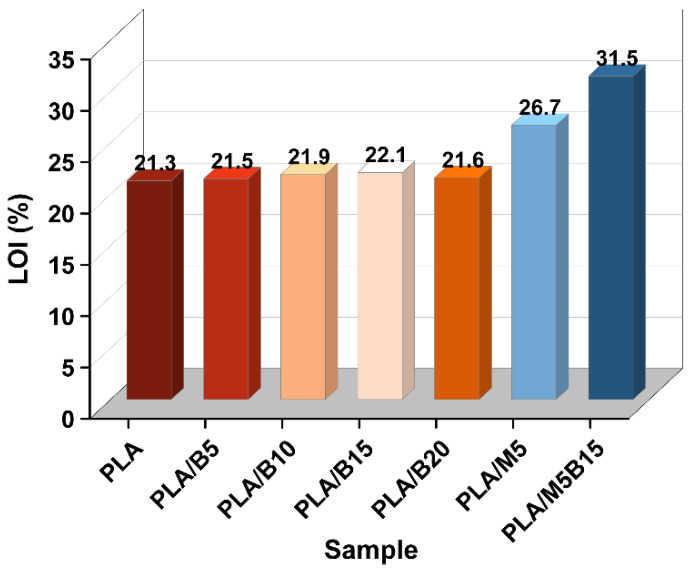
The limiting oxygen index (LOI) results of PLA composites.

**Figure 2:**
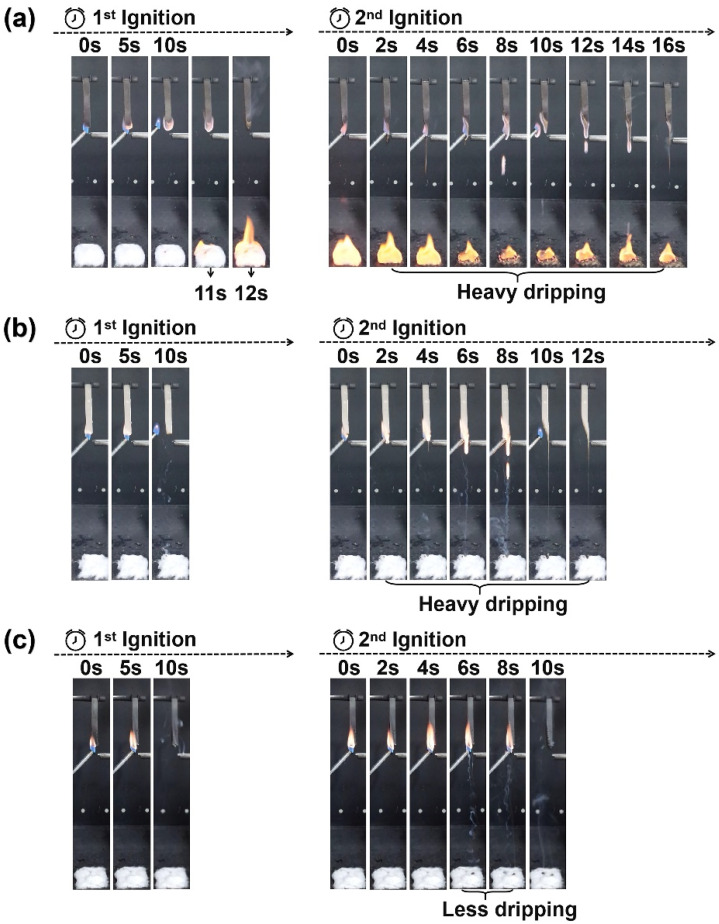
The video screenshots of (**a**) PLA/B15, (**b**) PLA/M5, and (**c**) PLA/M5B15 during UL-94 tests.

The flame retardancy of the composites was investigated by LOI and UL-94 tests. The results are given in Figure 1 and Figure 2 and Table 3. Pure PLA was easily flammable, showed severe burning and heavy dripping during the combustion process, and its LOI was only 21.3%. With the addition of BPP from 5 to 15 wt %, the LOI of the PLA composites improved from 21.5 to 22.1%. Nevertheless, the LOI of PLA/B20 exhibited a relatively lower value at 21.6%, which can be interpreted as the “candle wick effect”, in that the excessive addition of BPP was more likely to create a continuous mass path, and the larger thermal conductivity of BPP making the PLA composite more flammable [30]. With the addition of 5 wt % MCAPP, the LOI of the PLA/M5 increased to 26.7%. From UL-94 test, more details about ignition and dripping were provided. It can be found that PLA/B15 began to drip at 11 s and extinguished at 12 s after the first ignition and had heavy dripping from 2 s to 16 s after the second ignition, which passed the UL-94 V-2 rating. The self-extinguishing time of PLA/M5 was advanced, which may be attributed to the protective effect of silica shell [31]. However, the dripping phenomenon of PLA/M5 was still serious, especially after the second ignition. When MCAPP and BPP were combined with PLA matrix, the flame retardancy was greatly improved. The introduction of 15 wt % BPP to PLA/M5 significantly increased the LOI from 26.7% to 31.5% of the new composite (PLA/M5B15), obtaining UL-94 V-0 test. From Figure 2c, PLA/M5B15 immediately extinguished without any dripping after the first ignition, and only had significantly less dripping after the second ignition, which was obviously different from the heavy dripping problem existing in the PLA/B15 and PLA/M5. These results confirmed that the addition of 15 wt % BPP and 5 wt % MCAPP ameliorated the flame retardancy of the composite, and enhanced the abilities of self-extinguishing and anti-dripping.

**Figure 3:**
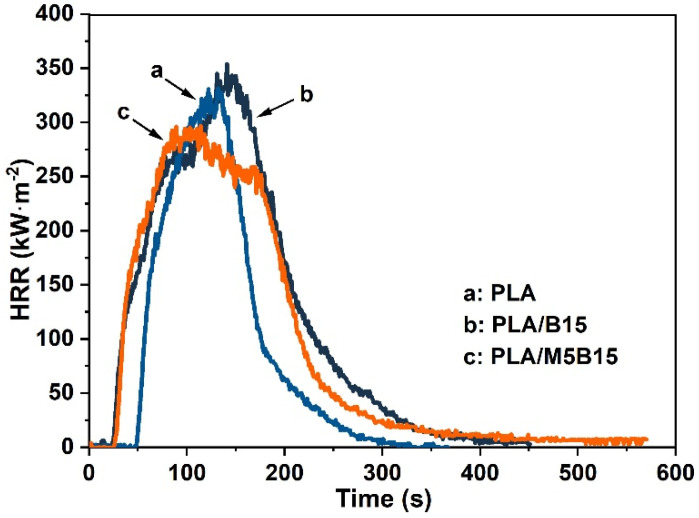
The heat release rate (HRR) curves of PLA, PLA/B15, and PLA/M5B15 composites.

To further investigate the enhancement of MCAPP on flame retardancy properties of PLA/B15 composites, PLA, PLA/B15, and PLA/M5B15 were tested by CCT. The curves of the heat release rate (HRR) were shown in Figure 3, and the time to ignition (TTI), peak heat release rate (pHRR), time to pHRR (tpHRR), average HRR (av-HRR), peak CO_2_ yield (pCO_2_y), and peak CO yield (pCOy) was illustrated in Table 4. From Figure 3 and Table 4, the pHRR of PLA was 331.4 kW·m^−2^. When 15 wt % BPP was added, the pHRR for PLA composite increased to 353.8 kW·m^−2^. By comparison, the addition of 5 wt % MCAPP and 15 wt % BPP to PLA decreased the pHRR value to 296.7 kW·m^−2^, reduced by 16.1% compared with that of PLA/B15. Furthermore, the addition of 5 wt % MCAPP shortened the tpHRR, meaning that MCAPP made a burning advance. Meanwhile, the av-HRR of PLA/M5B15 was the lowest (91.4 kW·m^−2^) compared to that of PLA and PLA/B15. It was worth mentioning that the pCO_2_y of PLA/M5B15 (6.3 kg·kg^−1^) was markedly lower than that of PLA/B15 (13.3 kg·kg^−1^), and the pCOy of PLA/M5B15 was just 0.02 kg·kg^−1^, which meant the addition of BPP and MCAPP can inhibit the production of CO_2_ and CO. The CCT results exhibited better flame retardancy performance of PLA/M5B15.

### 3.2. Thermal Properties of PLA Composites

**Figure 4:**
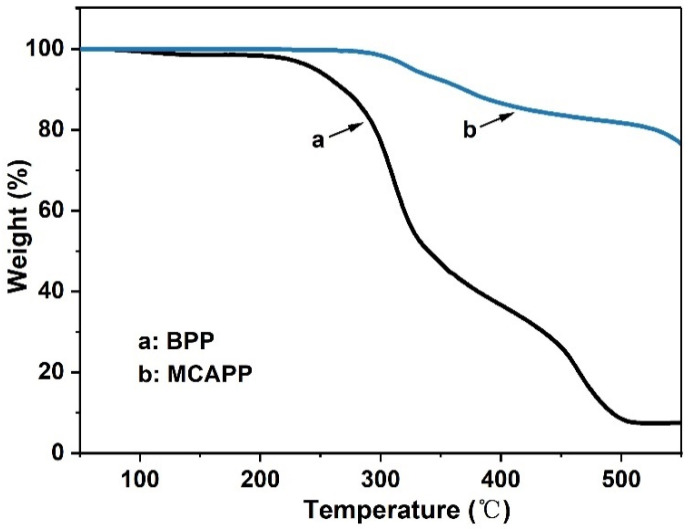
The thermogravimetric analysis (TG) curves of MCAPP and BPP.

**Figure 5:**
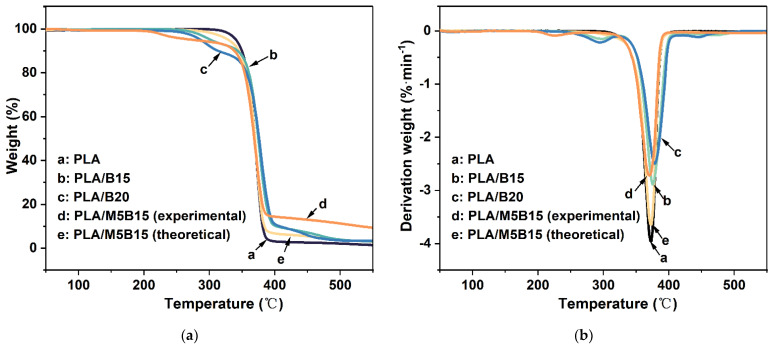
The TG (**a**) and differential thermogravimetric (DTG) (**b**) curves of PLA composites.

TG and differential thermogravimetric (DTG) curves were used to investigate the thermal properties of the composites, the results are shown in Figure 4 and Figure 5, and the related parameters are shown in Table 5. Figure 4 presents the TG curves of MCAPP and BPP. BPP degraded in multiple steps, the first mass loss appearing before 170.0 °C was mainly due to the evaporation of water, and the second mass loss was assigned to the decomposition of hemicellulose and cellulose occurring from 200.0 to 350.0 °C [32,33]. The pyrolysis of lignin was the principal cause of the last mass loss in the range of 380.0–500.0 °C [34]. The decomposition of MCAPP primarily included two steps. The first step at 320.0–370.0 °C, MCAPP released some volatile products such as ammonia and moisture and formed a crosslinked polyphosphoric acid layer; in the second step at around 500.0 °C, some MCAPP dehydrated to form phosphorus pentoxides. At elevated temperatures, the SiO_2_ gel on the MCAPP can form a compact silica shell, preventing the degradation of the APP [35]. Pure PLA presented one-step decomposition, 5 wt % weight loss temperature (T_5%_) and the maximum decomposition temperature (T_max_), respectively, at 341.0 °C and 373.3 °C, and the char residue was only 1.44 wt % at 550.0 °C (Figure 5). For PLA/B15, the addition of BPP led to a decrease in T_5%_, which was ascribed to the decomposition of hemicellulose and cellulose in BPP. Moreover, the char residue of PLA/B15 increased to 3.26 wt %, proving that the sufficient fixed carbon content in BPP promoted the carbonization of the PLA composite. A similar trend had also been obtained by other researchers [36,37,38]. With the addition of 20 wt % BPP, the combustion process was accelerated owing to the “candle wick effect”, but the lignin contained in BPP exhibited inherent heat resistance, slowing down the decomposition of PLA/B20 at high temperature, resulting in lower maximum mass loss rate (R_max_) (2.5%·min^−1^), and the char residue of PLA/B20 was relatively more than that of PLA/B15 [39]. As to PLA/M5B15, the T_5%_ transferred to a lower temperature at 275.0 °C, meaning that MCAPP catalyzed the degradation of BPP at low-temperature regions. This early degradation was mainly attributed to the formation of acidic phosphorus species from MCAPP, and was slightly influenced by the acidic characteristics of the silica gel [40]. However, the R_max_ of PLA/M5B15 (2.7%·min^−1^) was lower than PLA/B15 (2.9%·min^−1^) and PLA (4.0%·min^−1^), and its char residue was significantly increased to 9.32 wt % at 550.0 °C, which was much more than the others, demonstrating that PLA/M5B15 possessed the optimal thermal stability under the elevated temperatures. To further expose the interaction between MCAPP and BPP, Figure 5 showed the theoretical TG and DTG curves of PLA/M5B15 calculated by the weighted data of MCAPP, BPP, and PLA. It can be discovered that there were many differences between the theoretical and experimental curves of PLA/M5B15. The first mass loss does not appear in the theoretical curve, and the R_max_ of the theoretical curve is 3.6%·min^−1^ while the experimental curve was lower. Furthermore, the experimental curve showed a higher char residue than the theoretical one (3.00 wt %). These phenomena demonstrated there were some synergistic effects between MCAPP and BPP. Analogous results can be seen in the incorporation of other flame retardants into the PLA matrix, in which the acid released from pyrolysis can promote natural fiber intermolecular dehydration and charring, resulting in the advance of thermal decomposition and the increase of char residue formation [9,12]. In addition, the presence of silica-gel facilitates the dehydration of BPP to form stable carbonaceous and inorganic residues [41,42].

### 3.3. Morphology of the Char Residues

**Figure 6:**
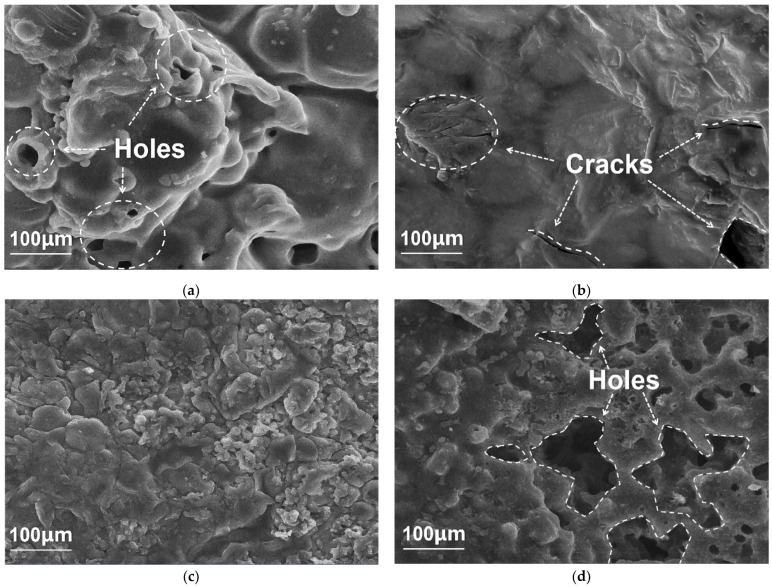
The scanning electron microscopy (SEM) photos of the residue chars of (**a**) PLA/B15, (**b**) PLA/M5, (**c**) PLA/M5B15, and (**d**) PLA/B20.

The morphology of char residues of PLA/B15, PLA/M5, PLA/B20, and PLA/M5B15 was observed by SEM, which were obtained after the heat treatment by muffle furnace at 350 °C for 10 min, and the photos are shown in Figure 6. As depicted by Figure 6a, PLA/B15 exhibited many holes on the surface of the char residue, which cannot provide sufficient protection to the matrix. The char residue of PLA/M5 displayed a relatively flat surface, which can be ascribed to the unreacted groups of Si in MCAPP, reacting with the terminal hydroxyl of PLA, ameliorating the interfacial interaction and boosting the solid phase activity [43]. Nevertheless, there were still many cracks on the surface which can lead to connection between the matrix and heat. It can be seen from Figure 6c that the char residue of PLA/M5B15 had an unsharp phase boundary, and a more continuous surface without obvious holes or flaws. In addition, the laminar agglomerated char residue distributed on the surface of PLA/M5B15 acted as an extra cover, while no improvement in char residue was found in PLA/B20 (Figure 6d) with the same loading. During combustion, MCAPP can work as a catalyst in the pyrolysis process and promote the charring of the PLA composites. On the basis of the agglomerated char layer, the protective effect on the substrate can be activated by blocking the propagation of heat and flammable gases, thus the flame retardancy of the composite was further reinforced.

### 3.4. Analysis of Pyrolysis Gaseous Products

**Figure 7:**
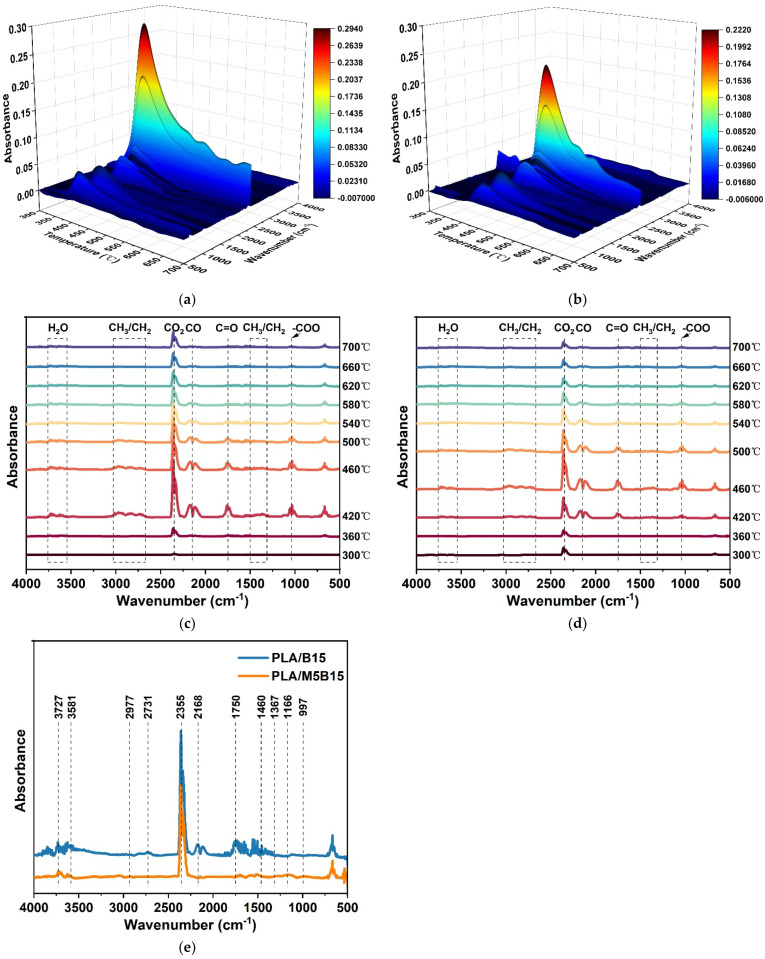
3D thermogravimetric analysis-infrared spectrometry (TG-IR) spectra of (**a**) PLA/B15 and (**b**) PLA/M5B15 under air atmosphere. IR spectra of PLA/B15 and PLA/M5B15 at (**c**,**d**) various temperatures and (**e**) 360 °C.

TG-IR was performed to further investigate the pyrolysis gaseous products during the thermal degradation. Figure 7 showed the TG-IR spectra of PLA/B15 and PLA/M5B15. In the spectra, the band in the range of 3800–3500 cm^−1^ was assigned to H_2_O [44]. The peaks at around 2977 and 2731 cm^−1^ were mainly caused by the asymmetric and symmetric vibration of saturated hydrocarbon (CH_3_/CH_2_), and those at 1460 and 1367 cm^−1^ were due to its deformation vibration [45]. The peak at 2355 cm^−1^ was attributed to the absorbance of CO_2_, while the peak at 2168 cm^−1^ was associated with CO. The band at 1750 cm^−1^ was ascribed to carbonyl compounds (C=O), and the peak at 1026 cm^−1^ was due to the aliphatic ester group (-COO). During the pyrolysis process, H_2_O and CO_2_ were released from the dehydration process, and the CH_3_/CH_2_, C=O, and -COO were generated by the decomposition of PLA and BPP. By comparison, the intensity of PLA/M5B15 was much lower than that of PLA/B15, indicating that the introduction of MCAPP inhibits the pyrolysis of the composites. More interestingly, the comparatively weak peaks appeared at around 1166 cm^−1^ and 997 cm^−1^ in the spectra of PLA/M5B15, which were assigned to the phosphorus-containing compounds (P-O-C) and NH_3_ originated from the thermal decomposition of MCAPP [46,47,48]. 

**Figure 8:**
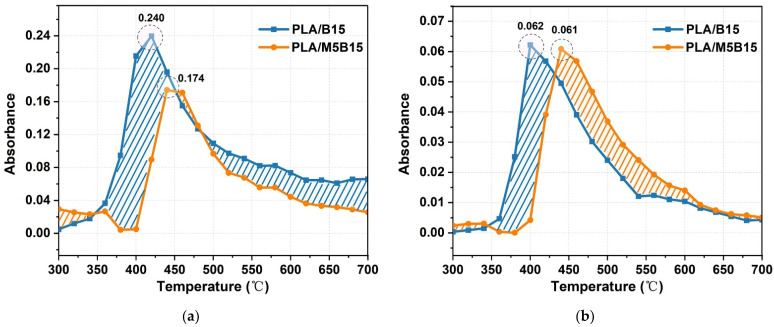

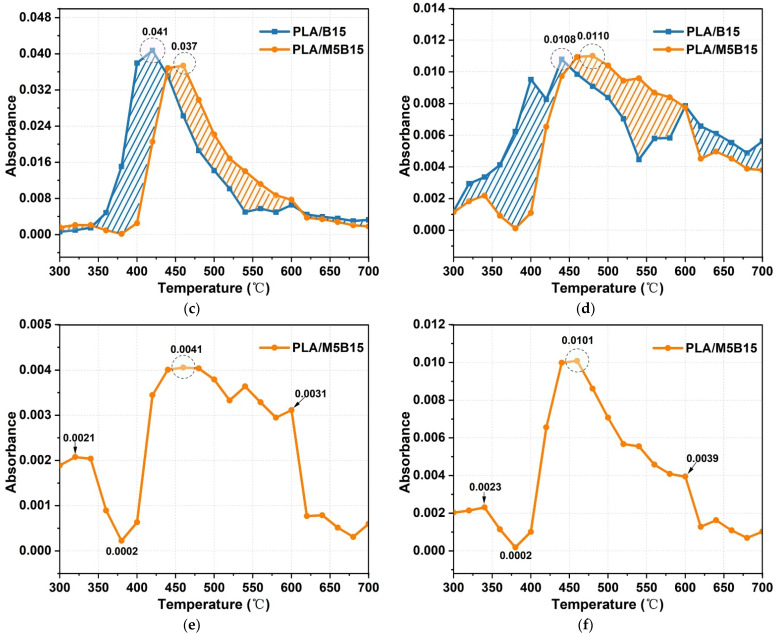
Absorbance of pyrolysis gaseous products for PLA/B15 and PLA/M5B15: (**a**) CO_2_; (**b**) CO; (**c**) C=O; (**d**) CH_3_/CH_2_; (**e**) NH_3_; (**f**) P-O-C.

Figure 8 reveals the absorbance intensity of typical pyrolysis gaseous products for PLA/B15 and PLA/M5B15 during the main degradation process, and the shaded part reflects the difference in absorption intensity at the same temperature. From Figure 8a, the release of CO_2_ of PLA/B15 can be divided into the initial release stage (300–360 °C) and mass release stage (380–420 °C), which reached the maximum absorbance intensity of 0.24 at around 420 °C. Compared to PLA/B15, the intensity of PLA/M5B15 exhibited an increase at the initial release stage, meaning that the degradation process occurred earlier than that of PLA/B15. Nevertheless, the mass release stage of PLA/M5B15 was delayed, and the maximum value of CO_2_ was reduced to a lower value. From the shadow of spectra, it was obvious that the total absorbance intensity of CO_2_ of PLA/M5B15 was considerably lower than PLA/B15. A similar occurrence can be observed in the intensity curves of CO and C=O. The reduced absorption intensities of PLA/M5B15 compared to those of PLA/B15, demonstrated that the pyrolysis gaseous products were suppressed by the “barrier effect” of the char layers. However, a weak increase in the maximum absorbance intensity of CH_3_/CH_2_ with the introduction of MCAPP can be seen in Figure 8d. At the temperature region of 300–450 °C, the PLA/M5B15 had a significantly lower intensity of CH_3_/CH_2_ than PLA/B15, and then displayed a relatively higher maximum absorbance intensity in the regions of 460–600 °C, even though the corresponding temperature was still delayed. That phenomenon can be interpreted as the massive release of non-flammable volatile products of NH_3_ from the pyrolysis of MCAPP, and was consistent with the regions of high absorption intensity of NH_3_ (Figure 8e). The existence of NH_3_ exerted flame retardant effects by diluting the concentration of the flammable gases around the composite, absorbing heat energy, and inhibiting the combustion of the substrate [47]. As depicted in Figure 8f, P-O-C had an apparent intensity in the region of 400–620 °C, generated during the degradation of the PLA/M5B15, which can terminate the chain reaction, capture OH· and H· and prevent the matrix from further heating [48]. Consequently, the interactions between BPP and MCAPP acted as a flame retardant owing to the “barrier effect” of protective char residue, delaying the temperature of the maximum absorbance intensity of CO_2_, CO, C=O, and CH_3_/CH_2_, and reducing the total release of gaseous products. Meanwhile, the release of incombustible gas (NH_3_) was beneficial to diluting the flammable volatiles, and the appearance of phosphorus-containing compounds radicals (PO) exerted flame retardant effect via capturing the combustible free radicals such as OH· and H in the gas phase, and therefore the combustion can be retarded.

## 4. Conclusions

Nowadays, natural materials are increasingly being used as biomass carbonization agents in flame retardant fields, preparing an efficient, cheap, and environmentally friendly flame retardant. In this study, the cheap natural material BPP was used to prepare PLA/BPP composites, and the flame retardancy was enhanced by introducing MCAPP. The results showed that the LOI of PLA contained 15 wt % BPP (22.1%) and passed the UL-94 V-2 rating. Moreover, with the introduction of 5 wt % MCAPP and 15 wt % BPP, the PLA composite had a higher LOI value of 31.5%, and reached the UL-94 V-0 rating, with self-extinguishing and anti-dripping abilities. The cone calorimetry results showed that PLA/M5B15 had a lower peak heat release rate (296.7 kW·m^−2^), which decreased by 16% more than that of the PLA/B15 composite. TG results indicated that PLA/M5B15 composite showed the highest char residue (9.32 wt %), meaning that the synergistic effect between MCAPP and BPP imparts better thermal stability to the composite. According to the SEM and TG-IR results, the addition of MCAPP and BPP accelerates the generation of high-quality char residue which is helpful in protecting the matrix from further burning, inhibiting the production of CO_2_, CO, C=O. Furthermore, the presence of NH_3_ and P-O-C can exert the flame retardant effect in the gas phase by diluting the flammable volatiles and capturing the combustible free radicals like OH· and H·. This work provides an effective strategy to enhance the flame retardancy of novel PLA/BPP composites and offers a new way to use agricultural wastes like banana peel.

## Figures and Tables

**Table 1:** The formulations of poly(lactic acid) (PLA) and PLA composites.

Samples	Component (wt %)		
	PLA	BPP	MCAPP
PLA	100	0	0
PLA/B5	95	5	0
PLA/B10	90	10	0
PLA/B15	85	15	0
PLA/B20	80	20	0
PLA/M5	95	0	5
PLA/M5B15	85	15	5

**Table 2:** Determination basis for vertical combustion test (UL-94) rating according to the GB/T 2408-2008 standard.

Determination Basis	Rating		
	V-0	V-1	V-2
Single specimen afterflame time (t_1_ and t_2_) ^1^	≤10 s	≤30 s	≤30 s
Total afterflame time for a set of samples (t_f_)	≤50 s	≤250 s	≤250 s
Afterflame time plus afterglow time of single specimen after second ignition (t_2_ + t_3_)	≤30 s	≤60 s	≤60 s
Whether the afterflame and/or afterglow spread to the fixture	No	No	No
Whether the flame particles or dripping ignite the cotton	No	No	Yes

^1^ t_1_/t_2_ is the afterflame time after removing the heat source at the first/second ignition.

**Table 3:** The UL-94 test results of PLA and PLA composites.

Samples 1	UL-94		
	Ignition of Cotton	Dripping ^1^	Rating
PLA	Yes	Y/-	No rating
PLA/B5	Yes	Y/Y	V-2
PLA/B10	Yes	Y/Y	V-2
PLA/B15	Yes	Y/Y	V-2
PLA/B20	Yes	Y/Y	V-2
PLA/M5	No	Y/Y	V-0
PLA/M5B15	No	N/Y	V-0

^1^ N/Y corresponds to No/Yes for dripping in the first/second flame application.

**Table 4:** Cone calorimeter test (CCT) data of PLA, PLA/B15, and PLA/M5B15 composites.

Samples	TTI (s)	pHRR (kW·m^−2^)	tpHRR (s)	av-HRR (kW·m^−2^)	pCO_2_y (kg·kg^−1^)	pCOy (kg·kg^−1^)
PLA	54	331.4	131	109.8	29.2	0.99
PLA/B15	29	353.8	140	127.3	13.3	0.04
PLA/M5B15	31	296.7	113	91.4	6.3	0.02

**Table 5:** Related thermal degradation parameters of PLA and PLA composites.

Samples	T_5%_ (°C)	T_max_ (°C)	R_max_ (°C)	Residue (wt %)
PLA	341.0	373.3	4.0	1.44
PLA/B15	301.7	375.0	2.9	3.26
PLA/B20	286.7	378.3	2.5	3.33
PLA/M5B15 (experimental)	275.0	370.4	2.7	9.32
PLA/M5B15 (theoretical)	328.5	373.3	3.6	3.00

## Data Availability

Not applicable.
